# Cholesterol synthesis inhibitors protect against platelet-activating factor-induced neuronal damage

**DOI:** 10.1186/1742-2094-4-5

**Published:** 2007-01-18

**Authors:** Clive Bate, Louis Rumbold, Alun Williams

**Affiliations:** 1 Department of Pathology and Infectious Diseases, Royal Veterinary College, Hawkshead Lane, North Mymms, Herts, AL9 7TA, UK

## Abstract

**Background:**

Platelet-activating factor (PAF) is implicated in the neuronal damage that accompanies ischemia, prion disease and Alzheimer's disease (AD). Since some epidemiological studies demonstrate that statins, drugs that reduce cholesterol synthesis, have a beneficial effect on mild AD, we examined the effects of two cholesterol synthesis inhibitors on neuronal responses to PAF.

**Methods:**

Primary cortical neurons were treated with cholesterol synthesis inhibitors (simvastatin or squalestatin) prior to incubation with different neurotoxins. The effects of these drugs on neuronal cholesterol levels and neuronal survival were measured. Immunoblots were used to determine the effects of simvastatin or squalestatin on the distribution of the PAF receptor and an enzyme linked immunoassay was used to quantify the amounts of PAF receptor.

**Results:**

PAF killed primary neurons in a dose-dependent manner. Pre-treatment with simvastatin or squalestatin reduced neuronal cholesterol and increased the survival of PAF-treated neurons. Neuronal survival was increased 50% by 100 nM simvastatin, or 20 nM squalestatin. The addition of mevalonate restored cholesterol levels, and reversed the protective effect of simvastatin. Simvastatin or squalestatin did not affect the amounts of the PAF receptor but did cause it to disperse from within lipid rafts.

**Conclusion:**

Treatment of neurons with cholesterol synthesis inhibitors including simvastatin and squalestatin protected neurons against PAF. Treatment caused a percentage of the PAF receptors to disperse from cholesterol-sensitive domains. These results raise the possibility that the effects of statins on neurodegenerative disease are, at least in part, due to desensitisation of neurons to PAF.

## Background

**Figure 1:**
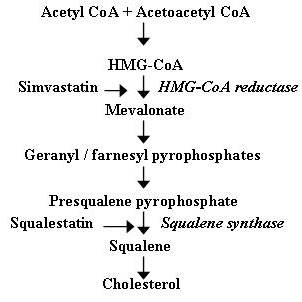
**Cholesterol biosynthesis pathway**. A biochemical pathway showing the major steps by which cholesterol is synthesised. Also shown are HMG-CoA reductase and squalene synthase, the enzymes that are inhibited by simvastatin and squalestatin respectively.

The hypothesis that brain cholesterol levels can affect the progression of Alzheimer's disease (AD) is now widely accepted [1]. One consequence of this hypothesis is the increasing interest in the use of statins as treatments for AD and other mild neurodegenerative disorders [2]. These drugs inhibit 3-hydroxy-3-methylglutaryl-co-enzyme A (HMG-CoA) reductase, the rate-limiting step in cholesterol production, and it is commonly thought that the main effects of statins are related to their cholesterol-lowering activity [3](Figure 1). While HMG-CoA reductase inhibitors reduce cholesterol levels, they also inhibit the production of isoprenoids such as the geranyl and farnesyl pyrophosphates [4]. Recently the effect of statins on non-sterol pathways such as isoprenoid production has been elucidated, and the role of isoprenoids on AD pathogenesis reviewed [5]. Such observations suggest that the effects of statins are not through cholesterol reduction alone. In the current study we compared the effects of simvastatin, a HMG-CoA reductase inhibitor, and squalestatin, an inhibitor of squalene synthase, which inhibits cholesterol production without affecting the production of non-sterol products [6] (Figure 1), on neurons.

Studies using platelet-activating factor (PAF) antagonists have indicated that PAF is involved in neuronal loss following human immunodeficiency virus infection [7], in kainic acid-induced epilepsy models [8] and in AD [9]. The effects of PAF are mediated via a specific receptor [10] which is coupled to cell-specific signalling pathways by G proteins [11]. In the current study we examined the effects of simvastatin and squalestatin on the sensitivity of primary cortical neurons to PAF and a variety of neurotoxins. We report that neurons treated with simvastatin or squalestatin demonstrate increased resistance to the otherwise toxic effects of PAF, but remain sensitive to neuronal injury induced by arachidonic acid or staurosporine. The protective effects of these drugs were associated with a significant reduction in neuronal cholesterol content, and the dispersal of the majority of PAF receptors from within detergent-resistant membranes.

## Methods

### Neuronal cultures

Primary cortical or cerebellar neurons were prepared from the brains of mouse embryos (day 15.5) after mechanical dissociation, cell sieving and isolation on histopaque (Sigma, Poole, UK). Neuronal precursors were plated (500,000 cells per well in 48 well plates coated with 5 μg/ml poly-L-lysine) in Hams F12 containing 5% fetal calf serum (FCS) for 2 hours. Cultures were shaken (600 r.p.m for 5 minutes) and non-adherent cells removed by 2 washes in phosphate buffered saline (PBS). Neurons were subsequently grown in neurobasal medium (NBM) containing B27 components (Invitrogen, Paisley, UK) for 7 days. Neurons were subsequently incubated with drug combinations for 24 hours before the addition of neurotoxins. The viability of neurons was determined 5 days later by the addition of 25 μM thiazolyl blue tetrazolium (MTT); neuronal survival was reported as a percentage of control cultures (untreated neurons).

### Neuronal membrane and lipid raft extracts

Treated neurons were scraped off plates and lysed at 1 × 10^6 ^cells per ml in distilled water containing 2 mM phenylmethylsulphonylflouride (PMSF). Membranes were isolated following physical disruption and a post nuclear supernatant was collected after centrifugation (300 × *g *for 5 mins). Neuronal membranes were collected by centrifugation (14,000 × *g *for 1 hr at 4°C). To dissociate lipid raft and non-raft membranes, total membranes were isolated as above and solubilised in a buffer containing 1% Triton X-100, 100 mM NaCl, 10 mM EDTA, 10 mM Tris-HCl, pH 7.8 and 5 mM PMSF. The mixture was incubated at 4°C for 1 hr; soluble material (bulk membrane) was collected after centrifugation (14,000 × *g *for 30 mins at 4°C). The insoluble pellet (lipid raft) was suspended in 100 mM NaCl, 10 mM Tris-HCl pH 7.8, 5 mM PMSF and 0.2% SDS. For immunoblot studies, pellets were dissolved in an extraction buffer containing 10 mM Tris-HCl, pH7.8, 100 mM NaCl, 10 mM EDTA, 0.5% Nonidet P-40, 0.5% sodium deoxycholate and 5 mM PMSF at 1 × 10^6 ^cells per ml. Sequential log 2 dilutions were made and 50 μl were added to duplicate wells. For western blot analysis samples were diluted 1 in 1 with Laemmli buffer (Bio-Rad) containing 2-mercaptoethanol and boiled for 5 minutes. 20 μl of each sample was subjected to electrophoresis on a 15% polyacrylamide gel and proteins were transferred onto a Hybond-P PVDF membrane (Amersham Biotech, UK) by semi-dry blotting. Membranes were blocked using 10% milk powder in Triz-buffered saline containing 0.2% Tween 20. PAF receptors were detected with polyclonal antibodies raised against a synthetic peptide containing amino acids 1–17 of the human PAF receptor (Cayman Chem, Ann Arbor, USA), a secondary anti-rabbit IgG extravidin conjugate followed by a biotin-alkaline phosphatase and visualised with 4-nitrophenol phosphate (Sigma). Membranes were also probed for β-actin using a mouse monoclonal antibody (Sigma, Dorset, UK). Protein concentration was determined using a micro-BCA protein assay kit (Pierce, Cramlington, UK) and amounts of cholesterol were measured using a fluorometric Amplex Red cholesterol assay kit with excitation at 550 nM and an emission detection at 590 nm (Invitrogen, Paisley, UK), according to the manufacturer's instructions. The amounts of PGE_2 _in cell extracts were determined using a competitive enzyme immunoassay kit (Amersham Biotech, Amersham, UK) according to the manufacturer's instructions.

### Enzyme-liked immunoassay (ELISA) for the PAF receptor

Total cell membranes or lipid raft fractions were suspended in carbonate buffer at 1 × 10^6 ^cells per ml, plated into 96 well immunoplates (50 μl per well) and incubated for 1 hour at room temperature to allow proteins to adhere. Non-specific binding sites were blocked by 10% milk powder and PAF receptors were detected by rabbit polyclonal antibodies to the human PAF receptor. Bound antibodies were detected with an anti-rabbit IgG alkaline-phosphatase conjugate and developed with 4-nitrophenol phosphate in diethanolamine buffer. Absorbance was measured on a microplate reader at 450 nM and PAF receptor content was calculated by reference to a standard curve. Samples were expressed as "units PAF receptor" where 100 units was arbitrarily defined as the amount of PAF receptor in 1 × 10^6 ^untreated cells. A standard curve was generated from this sample using sequential log 2 dilutions (range 100 to 1.56 units).

### Drugs

PAF (1-O-Hexadecyl-2-acetyl-*sn*-glycerol-3-phosphocholine) and simvastatin were obtained from Calbiochem (Nottingham, UK). Lyso-PAF (1-O-Hexadecyl-2-*sn*-glycerol-3-phosphocholine), arachidonic acid, mevalonate, squalene and staurosporine were obtained from Sigma (Poole, UK). Squalestatin was a gift from GlaxoSmithKline, Stevenage, UK.

### Statistical analysis

Comparison of treatment effects were carried out using one and two way analysis of variance techniques as appropriate.

## Results

### Treatment with simvastatin or squalestatin reduces the neurotoxicity of PAF

**Figure 2:**
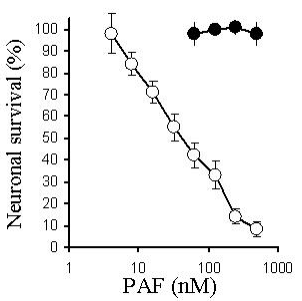
**PAF kills cortical neurons in a dose-dependent manner**. The survival of primary cortical neurons incubated with different concentrations of PAF (○) or lyso-PAF (●). Values shown are the mean average neuronal survival ± SD from 9 observations.

**Figure 3:**
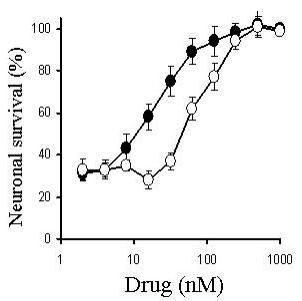
**Simvastatin and squalestatin protect neurons against PAF**. The survival of primary cortical neurons pre-treated for 24 hours with different concentrations of simvastatin (○) or squalestatin (●) prior to the addition of 100 nM PAF. Values shown are the mean average neuronal survival ± SD from 9 observations.

**Figure 4:**
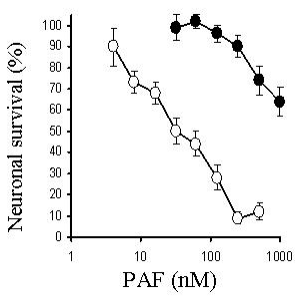
**Squalestatin-treated neurones are susceptible to high concentrations of PAF**. The survival of untreated primary cortical neurons (○) or neurons pre-treated for 24 hours with 100 nM squalestatin (●) prior to the addition of varying concentrations of PAF. Values shown are the mean average neuronal survival ± SD from 9 observations.

**Figure 5:**
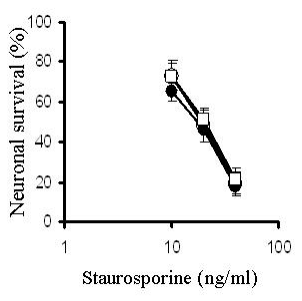
**Simvastatin and squalestatin do not protect neurons against staurosporine**. The survival of untreated primary cortical neurons (○) or neurons pre-treated for 24 hours with 500 nM simvastatin (□) or 100 nM squalestatin (●) prior to the addition of different concentrations of staurosporine. Values shown are the mean average neuronal survival ± SD from 9 observations.

**Figure 6:**
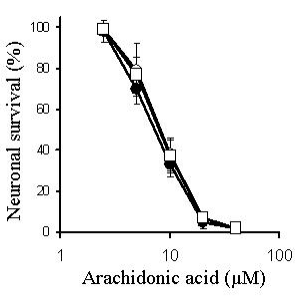
**Simvastatin and squalestatin do not protect neurons against arachidonic acid**. The survival of untreated primary cortical neurons (○) or neurons pre-treated for 24 hours with 500 nM simvastatin (□) or 100 nM squalestatin (●) prior to the addition of different concentrations of arachidonic acid. Values shown are the mean average neuronal survival ± SD from 9 observations.

The addition of PAF, but not lyso-PAF (an inactive metabolite of PAF), caused a dose-dependent reduction in the survival of primary cortical neurons; with an LD_50_~30 nM (Figure 2). To determine if cholesterol depletion affected neuronal responses to PAF, neurons were pre-treated with varying concentrations of squalestatin or simvastatin for 24 hours, prior to the addition of 100 nM PAF. Treatment of neurons with either squalestatin or simvastatin resulted in a dose-dependent increase in neuronal survival (Figure 3). While the concentration of simvastatin required to reduce the toxicity of PAF by 50% was 100 nM, the concentration of squalestatin required to provide a similar level of protection was 20 nM. Neurons treated with 500 nM squalestatin were not completely resistant to PAF, however the concentration of PAF required to kill 50% of squalestatin-treated neurons was 4 μM, approximately 100 times more than that required in untreated neurons (Figure 4). The effect of simvastatin or squalestatin on the amounts of cholesterol in primary cortical neurons was also determined. After 24 hours, the cholesterol content of neurons treated with 100 nM squalestatin was significantly less than that of untreated neurons (297 ng cholesterol/mg protein ± 32 v 496 ± 42, n = 9, P < 0.05). Similarly, the cholesterol content of neurons treated with 500 nM simvastatin was also significantly less than that of untreated neurons (331 ng cholesterol/mg protein ± 50 v 496 ± 42, n = 9, P < 0.05). To determine if cortical neurons treated with simvastatin/squalestatin were resistant to other neurotoxins, they were incubated with different concentrations of staurosporine or arachidonic acid. The survival of neurons treated with staurosporine, an activator of the ceramide pathway that induces apoptosis [12], was not significantly different after pre-treatment with either 100 nM squalestatin or 500 nM simvastatin (Figure 5). Similarly, the toxicity of arachidonic acid, a precursor to the production of neurotoxic prostaglandins [13], was not significantly different between untreated neurons and neurons treated with 100 nM squalestatin or 500 nM simvastatin (Figure 6).

### Squalene reverses the effects of squalestatin on neurons

To confirm that the protective effect of simvastatin and squalestatin were though inhibition of cholesterol synthesis, we sought to reverse the effects of these drugs with two precursors of cholesterol synthesis, mevalonate or squalene. We found no significant differences in the cholesterol content of untreated neurons and neurons treated with 100 μM mevalonate or with 50 μM squalene. While the addition of mevalonate or squalene reversed the effect of simvastatin on neuronal cholesterol levels, only squalene was able to reverse the effect of squalestatin on neuronal cholesterol (Table 1). The addition of either 100 μM mevalonate or 50 μM squalene alone did not affect the survival of neurons, nor did pre-treatment with mevalonate or squalene alter the survival of neurons subsequently treated with 100 nM PAF. While the addition of mevalonate or squalene reversed the protective effect of simvastatin, only squalene was able to reverse the protective effect of squalestatin (Table 2).

**Table 1:** Squalene reverses the effects of squalestatin on the amounts of cholesterol in neurons

**Treatment**	**Substrate**		
	**None**	**50 μM Squalene**	**100 μM Mevalonate**

	***Neuronal cholesterol content (ng/mg protein)***		

**None**	496 ± 42	472 ± 53	482 ± 53
**100 nM Squalestatin**	297 ± 32	475 ± 32	284 ± 29
**500 Nm Simvastatin**	331 ± 50	501 ± 46	485 ± 68

The amounts of cholesterol in primary cortical neurons treated for 24 hours with different combinations of either squalestatin or simvastatin together with either squalene or mevalonate as shown. Values shown are the mean average ± SD from 6 observations.

**Table 2:** Squalene reverses the protective effects of squalestatin

**Treatment**	**Substrate**		
	**None**	**50 μM Squalene**	**100 μM Mevalonate**

	***Neuronal survival (% of control)***		

**None**	31 ± 6	32 ± 4	30 ± 5
**100 nM Squalestatin**	95 ± 7	40 ± 9	93 ± 5
**500 nM Simvastatin**	94 ± 4	42 ± 9	34 ± 8

The survival of primary cortical neurons treated for 24 hours with combinations of either squalestatin or simvastatin together with either squalene or mevalonate as shown, and subsequently incubated with 100 nM PAF. Values shown are the mean average neuronal survival ± SD from 12 observations.

### Effect of squalestatin on the cellular location of the PAF receptor

**Figure 7:**
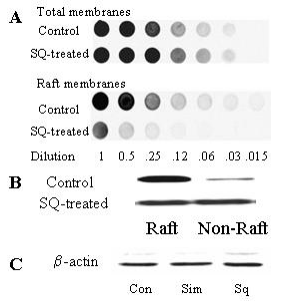
**Squalestatin alters the cellular location of the PAF receptor. **(A) Dotblots showing the amounts of PAF receptor in sequential dilutions (log 2 dilutions from neat) of extracts from untreated neurons and neurons treated for 24 hours with 100 nM squalestatin. (B) Western blot showing the amounts of PAF receptors in lipid raft/non-raft fractions from untreated neurons and neurons treated with 100 nM squalestatin. (C) Western blot showing the amounts of β-actin in cell extracts from untreated neurons (Con), or neurons treated with 500 nm simvastatin (Sim) or 100 nM squalestatin (Sq).

To determine if the protective effect of squalestatin was due to changes in the expression of PAF receptors, we examined membranes isolated from primary cortical neurons for the presence of PAF receptors. We were unable to distinguish any differences in the amounts of the PAF receptor in the total membrane fraction of untreated neurons and neurons treated with 100 nM squalestatin by dotblot (Figure 7). When an ELISA was used to quantify the amounts of PAF receptor in cells, no significant differences were observed between untreated and simvastatin-treated cells (100% ± 5 v 102% ± 7, n = 12, p > 0.05), or squalestatin-treated cells (100% ± 5 v 106% ± 9, n = 12, p > 0.05), showing that the resistance of these neurons to PAF was not due to a reduction in the number of PAF receptors. Since the PAF receptor is linked to various G-proteins [11] and many of the G-proteins are found in cholesterol sensitive lipid rafts [15] we questioned whether the PAF receptor may also be found in these domains. Fractionation of neuronal membranes revealed that in untreated neurons most of the PAF receptors reside within detergent-resistant membranes synonymous with lipid rafts. Following treatment with 100 nM squalestatin, a significant proportion of the PAF receptors were detected in the non-raft fraction of neuronal membranes (Figure 7). ELISA studies demonstrated significant differences between the amounts of PAF receptor detected in detergent-resistant membranes from untreated neurons and simvastatin-treated neurons (100% ± 9 v 38% ± 5, n = 12, p < 0.05) or squalestatin-treated neurons (100% ± 12 v 29% ± 4, n = 12, p < 0.05). The detergent-soluble fraction (non-raft membrane extract) from untreated neurons or neurons treated with 500 nM simvastatin or 100 nM squalestatin contained similar amounts of β-actin (Figure 7).

### Squalestatin reduces PAF-induced prostaglandin E_2 _production

**Figure 8:**
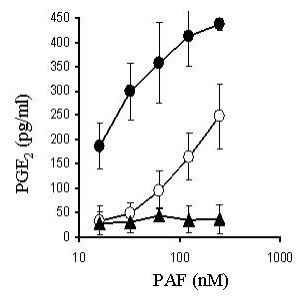
**Squalestatin reduces PAF-induced prostaglandin E**_2_**production**. The amounts of prostaglandin E_2 _(pg/ml) produced by untreated neurons incubated with different concentrations of PAF (●) or lyso-PAF (▲) or neurons pre-treated with 100 nM squalestatin and incubated with varying concentrations of PAF (○). Values shown are the mean prostaglandin E_2 _± SD from 9 observations.

Previous studies have shown that levels of prostaglandin E_2 _are elevated in Alzheimer's disease [14] and in this study we demonstrated that the addition of PAF caused a dose-dependent increase in prostaglandin E_2 _production. There was no significant difference in the production of prostaglandin E_2 _between untreated neurons and neurons treated with lyso-PAF. In neurons pre-treated with 100 nM squalestatin the effects of PAF on prostaglandin E_2 _production were significantly reduced (Figure 8).

## Discussion

In the present study, the effects of simvastatin or squalestatin on primary cortical neurons were examined. The amounts of cholesterol in neurons were significantly reduced by treatment with either simvastatin or squalestatin. The difference in the effects of these drugs is explained by their pharmacological targets (Figure 1). Cholesterol regulation within cells is under tight feedback control and is sensitive to the concentration of cholesterol in the endoplasmic reticulumn (ER). Reduced cholesterol in the ER results in production of HMG-CoA reductase [15] which catalyses mevalonate production and overcomes the effect of simvastatin (Figure 1) and in these studies the addition of exogenous mevalonate increased cholesterol production in simvastatin-treated cells. Since squalestatin inhibits squalene synthase, an enzyme further down the cholesterol synthetic pathway (Figure 1), the synthesis of new HMG-CoA reductase or the addition of exogenous mevalonate did not reverse the effects of squalestatin on neuronal cholesterol content. Although squalestatin is the more specific research tool, it does not cross the blood-brain barrier, which limits its therapeutic value.

Although PAF plays roles in the normal functioning of neurons, higher concentrations of PAF have been implicated in the neurotoxicity of epilepsy, ischaemia, human immunodeficiency virus infection, prion diseases and AD [16] PAF is not stored in a preformed state; rather it is synthesised in neurons in response to specific stimuli that activate phospholipase A_2_[17]. In this study, cortical neurons were killed by nanomolar concentrations of PAF, but not by lyso-PAF, a non-acetylated structural analogue of PAF that does not bind to the PAF receptor [18]. Pre-treatment with simvastatin or squalestatin protected neurons against the otherwise toxic effects of PAF; the IC_50 _for simvastatin was ~100 nM, and the IC_50 _for squalestatin was ~20 nM. We first tested simvastatin, a HMG-CoA reductase inhibitor, since it is one of the statins that penetrates the blood-brain barrier and is in clinical use [3]. However, although simvastatin reduces neuronal cholesterol content it also reduces the production of non-sterol products such as the isoprenoid precursors [4]. The modification of proteins by isoprenoids is essential for the function of a wide variety of proteins including the Ras-related G proteins[19]. Since some have argued that the effects of statins are mediated through inhibition of non-sterol products rather than cholesterol reduction [20]).) we also examined the effects of squalestatin, which inhibits squalene synthase, thus reducing cholesterol production without affecting the production of non-sterol products [6]. The observation that pre-treatment with squalestatin protects neurons against PAF suggests that cholesterol depletion is responsible for the observed neuroprotection. Furthermore, the protective effects of these drugs were reversed by the addition of squalene, a precursor of cholesterol synthesis that does not affect the production of non-sterol products (Figure 1). It is worth noting that neurons pre-treated with squalestatin were not completely resistant to PAF; approximately 50 times more PAF was required to kill squalestatin-treated cells than to kill untreated cells. Thus, it seems likely that the signalling pathways responsible for PAF-induced neurotoxicity remain intact in squalestatin-treated neurons but that the reduced cholesterol content of membranes hinders their activation.

Although pre-treatment with statins confers protection against amyloid-β peptides [21], prions [22] and PAF, it does not protect against all neurotoxic insults. Specifically, neurons treated with simvastatin or squalestatin remain sensitive to other neurotoxins including staurosporine and arachidonic acid. Staurosporine has been reported to have a number of effects that include activation of the ceramide pathway which is implicated in neuronal apoptosis [12]. We conclude that, in cortical neurons, the downstream pathways that lead to neuronal death activated by these neurotoxins are not sensitive to treatment with statins.

The effects of PAF are mediated via a specific receptor with seven transmembrane spanning segments [10]. We found no evidence that the protective effects of squalestatin or simvastatin were due to reduced amounts of the PAF receptor. Instead we identified effects of squalestatin and simvastatin on the location of PAF receptors within lipid rafts. In untreated neurons greater than 90% of the PAF receptors were found in lipid rafts. This observation is significant since the activation of downstream signalling pathways by PAF is dependent on interaction with pertussis toxin-sensitive G proteins [11], which also reside within lipid rafts [23]. The present results are consistent with the concept that PAF activates the PAF receptor in a lipid raft platform containing PAF receptors and G-proteins. The formation of some lipid rafts is cholesterol-dependent and therefore susceptible to treatment with cholesterol synthesis inhibitors [22]. Following treatment with squalestatin, significantly less PAF receptor was found within lipid rafts and more was found in the normal cell membrane. We propose that the PAF receptors outside lipid rafts fail to stimulate the G-proteins responsible for activation of downstream signalling pathways. This hypothesis is consistent with out observation that pre-treatment with squalestatin reduced PAF-induced prostaglandin E_2 _production.

## Conclusion

High concentrations of PAF are thought to contribute to neuronal damage in some neurodegenerative diseases. The current study demonstrates that inhibitors of cholesterol synthesis reduce the cholesterol content of neurons and greatly increases the resistance of these cells to PAF. This protective effect was associated with the dispersal of PAF receptors from within detergent-resistant membranes, or lipid rafts, and into the bulk cell membrane. The reduction of PAF receptors within detergent-resistant membranes was accompanied by a reduction in prostaglandin E_2 _production. We speculate that interactions between PAF and PAF receptors residing within the bulk cell membrane (outside lipid rafts) have a reduced capacity to stimulate downstream signalling pathways that lead to neuronal death. These results raise the possibility of using statins as adjuvant therapy for neurodegenerative diseases in which PAF has been implicated, such as ischaemia, stroke and AD. However, neuronal damage occurs via a variety of mechanisms *in vivo *and squalestatin- or simvastatin-treated neurons remain sensitive to other neurotoxins.

## Abbreviations

Alzheimer's disease (AD), platelet-activating factor (PAF),

## Competing interests

The author's declare that they have no competing interests.

## Authors' contributions

CB was responsible for the conception, planning and performance of experiments, and for writing this manuscript. LR prepared toxicity assays, western and dot blot analysis. AW contributed to the planning of experiments, interpretation of results and the writing of the manuscript.
